# GIS-Facilitated Seed Germination and Multifaceted Evaluation of the Endangered *Abies marocana* Trab. (Pinaceae) Enabling Conservation and Sustainable Exploitation

**DOI:** 10.3390/plants10122606

**Published:** 2021-11-27

**Authors:** Stefanos Hatzilazarou, Mohamed El Haissoufi, Elias Pipinis, Stefanos Kostas, Mohamed Libiad, Abdelmajid Khabbach, Fatima Lamchouri, Soumaya Bourgou, Wided Megdiche-Ksouri, Zeineb Ghrabi-Gammar, Vasiliki Aslanidou, Vasileios Greveniotis, Michalia A. Sakellariou, Ioannis Anestis, Georgios Tsoktouridis, Nikos Krigas

**Affiliations:** 1 Laboratory of Floriculture, School of Agriculture, Aristotle University of Thessaloniki, 54124 Thessaloniki, Greece; hatzilaz@agro.auth.gr (S.H.); skostas@agro.auth.gr (S.K.); vaslanid@agro.auth.gr (V.A.); 2 Laboratory of Natural Substances, Pharmacology, Environment, Modelling, Health and Quality of Life (SNAMOPEQ), Polydisciplinary Faculty of Taza, Sidi Mohamed Ben Abdellah University, B.P. 1223, Taza Gare, Taza 35000, Morocco; libiad001@gmail.com (M.L.); khamajid@hotmail.com (A.K.); fatima.lamchouri@usmba.ac.ma (F.L.); 3 Laboratory of Silviculture, School of Forestry and Natural Environment, Aristotle University of Thessaloniki, 54124 Thessaloniki, Greece; epipinis@for.auth.gr; 4 Laboratory of Ecology, Systematics and Biodiversity Conservation (LESCB), CNRST Labeled Research Unit N°18, Department of Biology, Faculty of Sciences, Abdelmalek Essaâdi University, B.P. 2121, M’Hannech II, Tetouan 93000, Morocco; 5 Laboratory of Biotechnology, Conservation and Development of Natural Resources (BCVRN), Department of Biology, Faculty of Sciences Dhar El Mahraz, Sidi Mohamed Ben Abdellah University, B.P. 1796, Fès-Atlas 30003, Morocco; 6 Centre de Biotechnologie de Borj-Cédria, Laboratoire des Plantes Aromatiques et Médicinales, B.P. 901, Tunis 2050, Tunisia; bourgousoumaya@yahoo.com (S.B.); ksouriwided@yahoo.fr (W.M.-K.); 7 Institut National Agronomique de Tunisie, Université de Carthage, 43 Avenue Charles Nicolle, Cité Mahrajène, Tunis 1082, Tunisia; zghrabi@yahoo.fr; 8 Laboratoire de Recherche Biogéographie, Climatologie Appliquée et Dynamiques Environnementales (BiCADE 18ES13), Faculté des Lettres des Arts et des Humanités de Manouba, Campus Universitaire de la Manouba, Université de la Manouba, Manouba 2010, Tunisia; 9 Institute of Industrial and Forage Crops, Hellenic Agricultural Organization Demeter, 41335 Larisa, Greece; vgreveni@mail.com; 10 Institute of Plant Breeding and Genetic Resources, Hellenic Agricultural Organization Demeter, 57001 Thermi, Greece; michsakellariou@yahoo.com (M.A.S.); ganestis3@gmail.com (I.A.); gtsok1@yahoo.co.uk (G.T.)

**Keywords:** sexual propagation, cold stratification, in situ, ex situ, plant endemism, Morocco, phytogenetic resources

## Abstract

In the frame of the sustainable use of neglected and underutilized phytogenetic resources, and along with numerous studies in *Abies* spp. due to the innate conservation value of fir forests, this research focused on the Moroccan endemic fir, *Abies marocana.* The aim was triple-fold: to assess its potential and dynamics in economic sectors for sustainable exploitation; to determine the ecological conditions in which the species naturally thrives; and to find the appropriate requirements for its successful seed germination. We sourced multifaceted evaluations for three economic sectors performed in three levels, using 48 attributes and eight criteria from previous studies of our own, and the relevant species-specific assessments are overviewed herein in detail. The species’ ecological profile was constructed using Geographical Information Systems (GIS) and open access data (Worldclim). Seed germination trials were performed to examine the effect of cold stratification (non-stratified, one- and two-months stratified seeds), the influence of four temperatures (10 °C, 15 °C, 20 °C, and 25 °C), and interactions thereof in relation to germination percentage (GP) and mean germination time (MGT). The experiments showed that the interaction of cold stratification and germination temperature has a strong effect on the GP and MGT of *A. marocana* seeds. A detailed GIS-derived ecological profile of the focal species was created in terms of precipitation and temperature natural regimes, enabling the interpretation of the seed germination results. The multifaceted evaluations reveal an interesting potential of the Moroccan fir in different economic sectors, which is mainly compromised due to extant research gaps, unfavorable conditions, and low stakeholder attraction. The findings of this study fill in extant research gaps, contribute to in situ and ex situ conservation strategies, and can facilitate the sustainable exploitation of this emblematic local endemic plant of northern Morocco.

## 1. Introduction

The genus *Abies* Mill. (Pinaceae) includes about 40 recognized species distributed in the temperate regions of the northern hemisphere [1]. Historically, members of the genus *Abies* have been a valuable phytogenetic resource for humans. Fir trees have been long-exploited for their timber and the production of paper [2], and studies suggest that *Abies* spp. has also been used in traditional medicine to heal people’s wounds [3]. In addition, *Abies* spp. has also been used widely in other sectors of the economy, such as the ornamental industry, with various fir species used traditionally as Christmas trees in different places [2]. 

The circum-Mediterranean firs form a distinct group of 10 native taxa and a natural hybrid, i.e., *Abies* × *borisii-regis* Mattf. [4]. Six fir species among them, i.e., *Abies cephalonica* Loundon, *A. cilicica* (Antoine and Kotschy) Carrière, *A. marocana* Trab., *A. nebrodensis* (Lojac.) Mattei, and *A. numidica* de Lannoy ex Carrière, are range-restricted in parts of the Mediterranean region. One of these, namely *A. marocana* (Moroccan fir), is a single-region local endemic species of Morocco which is confined to the Rif region, and it is classified as endangered, with a decreasing population [5]. This species could be considered as a relict tree species since it has endured a reduction of original distribution rather recently in Earth’s history, due to climate changes and/or anthropogenic activities [6,7]. Currently, only two populations of *A. marocana* exist in the Rif Mountains of Morocco; the first one is confined to Mt. Tazaout, and the second spreads over the Chefchaouen Mountains (the mountains of Sfiha Tell, Tissouka, Lakraa, Talassemtane, Bouslimane, Taloussisse, Fahs, and Kharbouch) included in the Talassemtane National Park, an area belonging to the Intercontinental Biosphere Reserve of the Mediterranean [5,6,8,9]. In general, this species is adapted to the humid Mediterranean climate with cold winters, tolerates frosts over long periods, and grows on dolomitic limestone substrates, between 1400 m and 2100 m above sea level [9]. 

The extant Moroccan fir forests are affected by several abiotic factors, including droughts, the average temperature of the warmest quarter, the maximum temperature of the warmest month [9,10], annual average temperatures, annual average precipitation, soil moisture, and lithology [11], biotic factors, such as competition with less demanding species, as well as anthropogenic activities, including logging and habitat alterations [6,8,12]. Previous works have reported population declines and the poor natural regeneration of *A. marocana* [6,13]. Historically, the exploitation of the Moroccan fir forests began in the first half of the twentieth century [8]. Since then, the natural distribution of *A. marocana* was reduced by 70%, due to massive logging, land clearing, and fires [9]. This trend is still decreasing the area occupied by *A. marocana* stands, from 5000 ha [6] to currently less than 4000 ha [9]. To date, an absence of middle-aged plants is reported due to selective cutting, and the overgrazing which is practiced across its range limits the potential of natural regeneration. The situation is different in the untapped fir forest of Tazaot, where an abundance of young plants has been reported [13]. Currently, *A. marocana* is protected in situ within the Talassemtane National Park, where no logging is carried out. Forest fires and Indian hemp cultivation [8], however, may still threaten the extant species range. 

In a wider context, the effects of climate change on *A. marocana* are, rather, perceived as moderate, due to the assumption that slow-growing conifers are able to regenerate under shelter if there are enough seedlings in mixed (*Abies marocana*–*Cedrus atlantica*) forests [10]. The structure, species composition, and competition in Moroccan fir forests could affect the response and resilience of *A. marocana* to climate change [10]. The competition with *Quercus ilex* L., *Q. faginea* Lam., and *Juniperus oxycedrus* L. could limit the regeneration of the Moroccan fir [12], while *A. marocana* and *Pinus nigra* J. F. Arnold-mixed forests seem to have a positive-to-neutral effect on *A. marocana* growth [10]. In the Tazaout forest, Moroccan firs regenerate more easily, and its stands are still dominant in the mixed formation with *Acer granatense* Boiss. and *Cedrus atlantica* (Endl.) Manetti ex Carrière, or when they co-occur with *Pinus pinaster* Aiton and *P. nigra*. However, 200 ha of Moroccan fir forest was burned in 2002, on the northern slope of Tazaout forest, and no regeneration has been observed there, probably due to the steep sloping and strong competition with other less-demanding species [8]. To resolve regeneration problems for this species in situ, deeper knowledge of *A. marocana* biology, as well as appropriate forest management actions, are required [10]. 

In general, to compile the necessary body of biological knowledge of threatened species (such as *A. marocana*), previous studies have highlighted the importance of seed germination studies both for the in situ and the ex-situ conservation of threatened plant resources [14,15,16,17,18]. Despite the fact that a large part of the Moroccan endemic flora includes hundreds of globally threatened plants [19], the number of local endemic taxa for which germination studies have been performed to date is still very low [20,21]. Currently, there are only a few germination studies related to some local endemic medicinal-aromatic plants of Morocco, such as *Thymus maroccanus* Ball., *Thymus broussonetii* Boiss [22,23], and *Origanum elongatum* (Bonnet) Emb. and Maire [15,24], and there is no relevant data for *A. marocana*. 

Conservation-wise, germination and propagation trials need to be species-specific and focused on threatened local endemic species to support and facilitate long-term conservation strategies, as well as effective sustainable exploitation strategies [18]. In the absence of published data concerning the seed germination of *A. marocana*, and in the framework of diversified seed dormancy reports for different *Abies* species [25,26], the study herein investigates, for the first time, the seed germination requirements of *A. marocana*, an emblematic local endemic tree of Morocco which is threatened by extinction and thus should be prioritized. The absence of relevant information for the targeted fir species makes its propagation by domestic forest authorities difficult, hindering conservation plans and restoration programmes and hampering its possible exploitation in various economic sectors. Concerning the latter, the promising potential and the feasibility of creating value chains for *A. marocana* in different economic sectors make its sustainable exploitation achievable [18,27,28]. In this context, an understanding of the life cycle of *A. marocana* and its seed germination is crucial for achieving both its effective in situ conservation and its successful ex situ propagation for conservation purposes and sustainable exploitation strategies. To this end, an ecological profiling using Geographical Information Systems (GIS) can provide significant insight regarding the abiotic environmental conditions that *A. marocana* requires in its natural habitats, and may inform whether these conditions can be exploited or can be reproduced to some extent in the man-made environment of the ex-situ conservation efforts, thus facilitating propagation, germination trails, and acclimatization [29,30]. In turn, information stemming from GIS-facilitated germination studies on seed storage, the needed pre-treatments, and the optimal temperatures for seed germination is very useful for in situ conservation efforts as well as for sustainable exploitation strategies. Consequently, in the present study, we generated the GIS ecological profile of *A. marocana* with the aim to examine the effect of cold stratification, and to evaluate the impact of temperature on its seed germination.

## 2. Results

### 2.1. Overview of the Potential of Abies marocana in Economic Sectors

#### 2.1.1. Ornamental-Horticultural Potential

**Figure 1:**
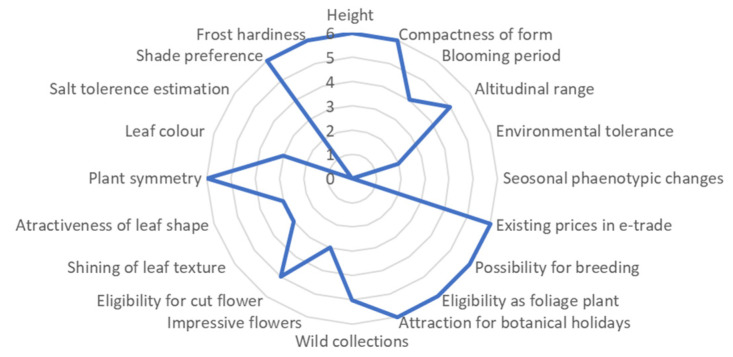
Evaluation of *Abies marocana* scored for 20 ornamental-horticultural attributes, reaching 72.5% of the optimum possible score, which is hierarchically ranked in the highest (>70%) general class; for details, see [18].

Among the 94 Moroccan endemics comparatively examined [18], the highest-evaluated taxon was *Abies marocana* (72.5%), showing an interesting general potential in the ornamental-horticultural sector. The scoring of *Abies marocana* is illustrated in Figure 1, profiting from high-scoring in most of the attributes examined. 

Concerning the potential in the subsectors of the ornamental-horticultural industry, *Abies marocana* scored 71.88% and ranked in the top three cases of local Moroccan endemics with the highest suitability for pot/patio plants (after *Salvia interrupta* subsp. *paui* and *Rhodanthemum hosmariense*). Although none of the local Moroccan taxa entered to the highest class of scores for home gardening suitability (>70%), *Abies marocana* received the highest score (68.83%) among them, and ranked first in above-average to high positions, followed by another seven Moroccan taxa. After *Salvia interrupta* subsp. *paui* and *Rhodanthemum hosmariense* ranked in top positions for landscaping eligibility (70.97% and 69.89% respectively), *Abies marocana* ranked in above-average to high positions (66.13%), followed by another eleven Moroccan taxa. Among Moroccan local endemics that are suitable for xeroscaping, *Abies marocana*, with 62.85%, ranked within the top 10 ones. 

#### 2.1.2. Medicinal-Cosmetic and Agro-Alimentary Potential

**Figure 2:**
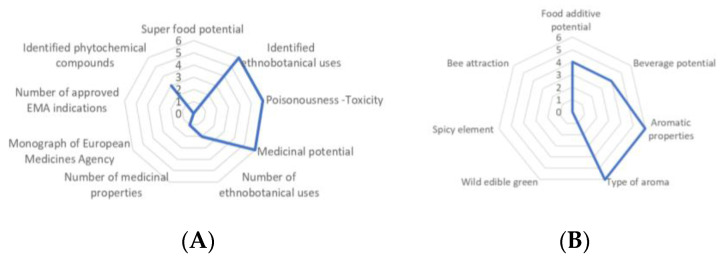
Evaluation of *Abies marocana* scored for nine medicinal attributes (**A**) and for seven agro-alimentary attributes (**B**), reaching 44.44% and 47.62% of the optimum possible scores, respectively; these scores hierarchically ranked it in corresponding lower to average classes [20,28], respectively.

*Abies marocana*, scoring 44.44% in the medicinal-cosmetic sector (Figure 2A), was the third-highest evaluated taxon among the 94 local Moroccan endemics examined [27,28], showing a very interesting potential. 

Although *Abies marocana* scored 47.62% in the agro-alimentary sector, it was included among the top 15 cases of local Moroccan taxa, with interesting agro-alimentary potential due to the strong aromatic properties of the pleasant aroma of the sourced resin (Figure 2B).

#### 2.1.3. Feasibility and Readiness Assessments for Sustainable Exploitation

In terms of feasibility for sustainable exploitation (Level II evaluation based on 12 attributes) [18], *A. marocana* received the highest score (43.06%) among 94 local endemic plants of northern Morocco, which hierarchically ranked the taxon in the below-average to low class. 

The readiness timescale for value chain creation regarding *A. marocana* (Level III evaluation upon completion of eight criteria) was assessed as achievable in the long-term [18]. 

### 2.2. Ecological Profiling of Abies marocana

**Figure 3:**
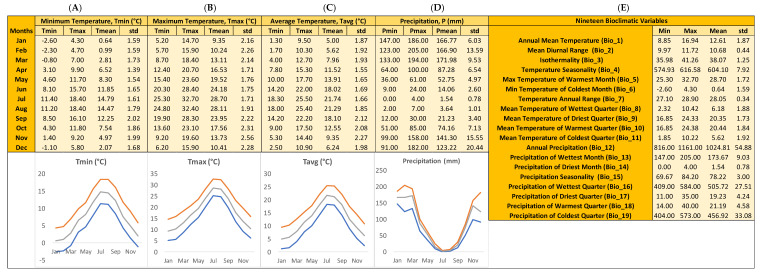
GIS-derived ecological profile across the natural distribution range of *Abies marocana* wild-growing populations in Morocco, based on 313 occurrence records. In each case (**A**–**E**), minimum, maximum, average, and standard deviation is shown. Line graphs illustrate the minimum (blue lines), maximum (orange lines), and mean (grey lines) monthly values for temperature (°C) and precipitation (mm). (**A**) Minimum temperatures per month (°C), (**B**) maximum temperatures per month (°C), (**C**) average temperatures per month (°C), (**D**) precipitation per month (mm), (**E**) values for 19 bioclimatic variables. All data were extracted from WorldClim version 2.1.

In an attempt to facilitate both conservation efforts and sustainable exploitation [17], a GIS ecological profile was generated in terms of temperature and precipitation regimes across the original sites, where *A. marocana* is wild-growing in the natural environment (Figure 3). 

#### 2.2.1. Temperature-Related Attributes 

The highest mean values of the average temperatures in the range of *A. marocana* (Figure 3) are peaked in mid- and late-summer, i.e., July (21.74 ± 1.66 °C) and August (21.29 ± 1.85 °C). During the autumn months, average temperatures start decreasing gradually, from 18.10 ± 2.12 °C in September to 9.35 ± 2.27 °C in November, and continue to reduce until mid-winter (5.00 ± 1.87 °C in January). By the end of winter (February), average temperatures start rising (5.62 ± 1.92 °C) and continue to increase gradually during the spring months, from 7.96 ± 1.93 °C in March to 13.91 ± 1.65 °C in May (Figure 3), reaching 18.02 ± 1.69 °C in early summer (June). Within this seasonal pattern, the minima of mean temperatures can be as low as −2.60 °C in January, and the maxima of mean temperatures can be as high as 32.70 °C in July (Figure 3). These temperature extremes represent the temperature limits in which the wild-growing *A. marocana* populations are adapted to thrive naturally, while mean diurnal range is 10.68 ± 0.44 °C and the annual temperature range is 12.61 ± 1.87 °C. These historical climatic data show no evidence for extreme low or high temperatures experienced by the *A. marocana* wild-growing populations. During the lowest average-temperature month (January), minimum mean temperature does not drop below 0 °C (T_mean_ of T_min_ = 0.64 ± 1.59 °C), while maximum mean temperature does not rise above 30 °C (T_mean_ of T_max_ = 28.7 ± 1.71 °C). 

#### 2.2.2. Precipitation-Related Attributes 

Historical precipitation records suggest a strong seasonal raining pattern in the natural range of *A. marocana* (Figure 3). The highest mean values of precipitation occur naturally at the beginning of spring (March), with 171.98 ± 9.53 mm after a four-month period of high precipitation from the end of autumn and during winter (Figure 3), i.e., from November (141.30 ± 15.55 mm) to February (166.90 ± 13.59 mm). In mid-spring (April), precipitation starts declining from 87.28 ± 6.54 mm to 1.54 ± 0.78 mm during the driest month (July). After mid-summer, precipitation rises again (Figure 3), mainly in September (21.23 ± 3.40 mm) and October (74.16 ± 7.13 mm).

### 2.3. Seed Germination Tests

The viability of the *A. marocana* seeds used in the experiments was low: 39 ± 8.24% (average of the four replications, S.D.), as indicated by the tetrazolium test.

Cold stratification (CS) and temperature, as well as the interaction thereof, significantly affected both the germination percentage (GP) and the mean germination time (MGT) of *A. marocana* seeds (Table 1). In the presence of a significant interaction, it was considered that the separate interpretation of the main effects was of inferior importance. In non-stratified seeds, the seeds incubated at 15 °C exhibited higher GP than seeds incubated at 10 °C, whereas no significant difference was observed in the GPs among seeds incubated at 10, 20, and 25 °C (Table 2). In cold-stratified seeds for one or two months, no significant difference among the incubation temperatures was observed in the GPs. Furthermore, in seeds incubated at 15, 20, or 25 °C, no significant difference was observed in the GPs between non-stratified seeds and seeds stratified for one or two months. However, in seeds incubated at 10 °C for the period of one month, CS resulted in a higher GP than non-stratified seeds, whereas no significant difference was observed in the GPs among seeds stratified for two months and non-stratified seeds (Table 2).

According to Table 2, a significant effect of CS on MGT was observed. The CS for one or two months significantly hastened the germination of *A. marocana* seeds. Particularly, at 10 °C, seeds stratified for two months exhibited the lowest MGT. In seeds incubated at 15, 20, or 25 °C, a lower MGT was observed in stratified seeds (regardless of the period) compared to the non-stratified ones, whereas no significant difference was observed between the two periods of CS examined. Furthermore, the seeds incubated at 10 °C for germination exhibited the highest MGTs, regardless of CS period.

**Figure 4:**
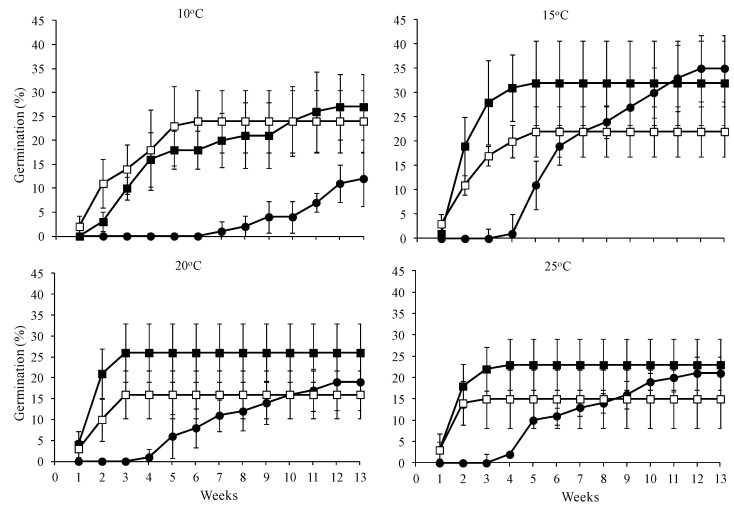
Effects of the cold-stratification period (CS) on the *Abies marocana* seed germination time course (● non-stratified, ■ one-month CS, and □ two-month CS) in seeds incubated at four constant temperatures (10, 15, 20, and 25 °C) under a 12-h light/12-h dark photoperiod.

When incubated at temperatures ≥15 °C, following a one- or two-month period of CS, germinated seeds were recorded on the seventh day after the sowing and the germination was completed, at the end of third week for seeds incubated at 20 and 25 °C, and two weeks later for seeds incubated at 15 °C (Figure 4). In non-stratified seeds, the germination started at the end of the fourth week at temperatures ≥15 °C and at the end of the seventh week at 10 °C (Figure 4).

## 3. Discussion

In the context of the sustainable use of phytogenetic resources and in the face of climate change, the Neglected and Underutilized Plant species (NUPs) are considered promising alternative crops if domesticated and sustainably used [18,31]. This study has explored, in a comprehensive way, and has assessed, in a multifaceted mode, whether the domestication procedure for *Abies marocana* can actually be achieved for its sustainable exploitation (see Section 4.1). Conservation-wise, this focal fir tree represents an emblematic local endemic of northern Morocco, which has been assessed as currently threatened by extinction, i.e., endangered with a decreasing population trend [5]. Therefore, intense research efforts are needed to obtain more information regarding its biological cycle if its extinction is to be prevented. In this way, the ecological profiling of *A. marocana* in terms of temperature and precipitation, as well as the first-ever germination tests presented herein, are considered contributions to fill such research gaps (see Section 4.2). 

### 3.1. Potential, Feasibility, and Readiness for Value Chain Creation

*Abies marocana* represents an important NUP of Morocco, which is interesting for applications in different economic sectors. According to the multifaceted evaluations exercised [18,27,28] and overviewed herein, *A. marocana* represents a promising and highly suitable species for the ornamental-horticultural sector, especially as a pot plant as well as for landscaping and home gardening applications [18]. Currently, *A. marocana* has recognized ornamental value and is currently traded worldwide by three nurseries over the internet, and distinct value chains are already extant [21]. This kind of electronic commerce, however, for such a unique floristic element of Morocco, is performed with no official permission granted by domestic authorities; it is subjected to sovereign rights due to the Nagoya protocol and may be further associated with conservation implications [21]. 

Almost the same interest applies for the medicinal-cosmetic sector, where *A. marocana* scored third in comparison to other local endemic NUPs of Morocco [28]. To date, comparative studies in members of the genus *Abies* report at least 277 compounds that have been isolated and identified across 19 species, reporting mostly terpenoids, flavonoids, lignans, phenols, and steroids [32]. Members of such categories of natural ingredients often find commercial applications in cosmetic products. Modern investigations in close *Abies* relatives report the isolation and identification of more than 90 constituents from *A. alba* seeds and cone scales, concluding that its seeds can be potentially exploited by the perfume industry [33]. Preliminary studies in *A. marocana* report a variety of diterpenoids, cadinanes, and cholestanes [32]. Yet, with limited available data to date, intense efforts and targeted phytochemical investigations are required to unveil the potentially valuable diversity of molecules and bioactive compounds that are probably naturally produced by *A. marocana*. 

*A. marocana* ranked almost average (43.06%) in terms of feasibility evaluation for sustainable exploitation [18]. However, when this score is partitioned in different attributes, two opposite trends became evident: (i) the relative tendency of score increases because of high attribute assessments (scores of five or six) related to endemism rarity, extinction risk status, effective ex situ conservation [20], and current extant horticultural experience with seed germination trials as presented herein, and (ii) the relative tendency of score decreases due to low attribute assessments (scores of zero or one) owing to the absence of protection status and few marketed commercial products [21], high water demands, the restricted availability of initial propagation materials, the absence of vegetative propagation techniques or cultivation protocols or extant cultivations [18]. If the above-mentioned gaps and weaknesses are filled by targeted applied research, there is no doubt that the feasibility assessment of *A. marocana* will be improved considerably, reaching those reported for some local endemics of Crete (Greece) that are currently under sustainable exploitation, i.e., *Sideritis syriaca* subsp. *syriaca* [34] and *Origanum dictamnus* [18].

The readiness timescale for the sustainable exploitation of *A. marocana* is assessed as achievable in the long-term [18] due to restrictions related with propagation materials, limited knowledge of propagation–cultivation techniques, compromised commercial interest, low stakeholder attraction, and discrepancies in implementation of the Nagoya Protocol in different countries. The absence of experience in the massive propagation and in the cultivation protocols for this species may strongly compromise any attempt at upscaling for its future exploitation and commercialization, and further implies that multidisciplinary and applied research is needed urgently (e.g., massive propagation, cultivation, cultivation practices, agro-processing, fertilization regimes, agronomical aspects, stakeholder and distribution channels attraction, etc.) to overcome existing barriers. Therefore, the value chain for *A. marocana* is considered as difficult to create in the short- or medium-term, and consequently, can only be achieved in long-term. This study, however, provides solid information regarding the seed propagation of *A. marocana*, thus facilitating the sustainable exploitation of this promising species in economic sectors. For many species, propagation from seeds is the most common and the cheapest method exercised in commercial nurseries [35]. In this fashion, it seems that there are good perspectives for sustainable exploitation if more research gaps are filled promptly and quickly. The speeding-along of sustainable exploitation strategies can also be achieved when similar successful paradigms at local scales are followed. For instance, *Argania spinosa* (L.) Skeels (Sapotaceae) has long been another NUP which—after targeted research by scientists, documentation, conservation efforts, and stakeholder attraction were all achieved within about a decade [27]—is currently receiving increased global appreciation in the international markets [36]. 

### 3.2. Seed Germination Requirements for Conservation and Sustainable Exploitation

For many species, propagation from seeds is the most common and the cheapest method used in commercial nurseries [35], creating heterogenous individuals due to species genetic diversity. Although this may be undesirable in some cases (e.g., the artificial selection of suitable genotypes for the production of specific metabolites), it is highly required during conservation efforts to maintain and promote the genetic diversity of the focal taxon. However, a major constraint to sexual propagation is the poor germination due to poor natural seed quality and seed dormancy. Indeed, high percentages of empty seeds are often observed in many *Abies* spp. [26]. A significant problem in such cases is the separation and the removal of seeds that do not contain an embryo (non-viable seeds), especially when these are filled in with resin. As frequently reported in the relevant literature, the seeds of most (if not all) members of the genus *Abies* exhibit some degree of dormancy, and therefore a period of CS is required to overcome it [25,37]. However, seed dormancy among different *Abies* spp. is quite variable, and the degree of dormancy determines the duration of CS needed to overcome this natural barrier [26]. Furthermore, a variation in seed dormancy among provenances and among different seedlots harvested in different years has been reported for some *Abies* spp. [25]. Due to this multiple variation in seed dormancy, a three-week period of stratification at 3–5 °C is suggested as a necessary step by ISTA [38] for seed germination in 10 *Abies* spp., whereas double germination tests are recommended for many species (including *A. pinsapo* Boiss., the closest species to *A. marocana*).

It is well-known that seeds of *Abies* spp. are usually of poor quality [39,40,41]. According to the results of the tetrazolium test performed in this study, the potential germination capacity of the seedlot of *A. marocana* sourced from wild habitats in Rif, Morocco and used in the germination experiments was as low as 39%. Possibly, the date of cone collection, the storage period (12 months) of seeds, or both of them resulted in a reduction in the viability and germinability of the seeds of *Abies marocana*. It has been reported that seed germinability of many *Abies* species is improved when seeds are collected as close as possible to seed dispersal [26]. Concerning the effect of seed storage on germination, the storage of *A. cephalonica* seeds in the dark, in sealed containers, at 7 ± 2 °C for one year resulted in a significant decrease in seed germinability [40]. Furthermore, the germination of *A.* ^x^ *borisii-regis* seeds is reduced after a one-year period of storage at 0 °C, compared to fresh seeds [41]. Similarly, low viability (<44%) has been reported in seeds from four Polish provenances of *A. alba* Mill. [42], and low germination percentages have been observed in seeds of four Indian provenances of *A. pindrow* (Royle ex D.Don) Royle [43]. 

In the present study, the germination and germination rate of *Abies marocana* non-stratified seeds varied along a temperature gradient, and its seed germination seemed to have a specific temperature requirement. *Abies marocana* seeds without any treatment germinated best at 15 °C, while a remarkable number of viable seeds failed to germinate at a lower (10 °C) temperature. In the literature, there are studies that recommend alternating temperatures of 30° with light for 8 h, and 20 °C with dark for 16 h, to achieve maximum seed germination in different *Abies* species [38], while some others note that species often have a specific temperature range for seed germination [37]. *Abies amabilis* (Douglas ex Loudon) J. Forbes seeds germinate better (although slower) under a 15:10 °C (day:night) temperature regime, regardless of the stratification treatment [44]. A constant temperature of 10 °C is probably the best temperature for the seed germination of *A. pindrow* from four provenances [43], as the highest GP and MGT is observed in this temperature (in contrast, the lowest GPs and MGTs were recorded at 25 °C). However, *A. cephalonica* seeds seem to germinate well over a wide temperature range (5–25 °C) without any pre-treatment, at both constant and alternating temperatures [45].

In our study, the CS of *A. marocana* seeds resulted in the loss of sensitivity to temperature. In cold-stratified seeds, regardless of duration, no significant difference in GPs among the incubation temperatures was observed. Apart from widening the range of temperatures for optimum germination, stratification resulted in more rapid germination of *A. marocana* seeds, thus confirming similar results found in previous studies for other *Abies* spp. [26,46,47]. In practical horticulture for sustainable exploitation, apart from high seed germination, uniform and rapid seed germination is equally significant in order to avoid environmental hazards within the nursery [36]. In general, the fastest rate of germination is usually observed at temperatures between 15 and 25 °C for the seeds of many plant species. However, the germination percentages of the cold-stratified seeds of *A. marocana* studied herein were found to be lower than those of viable seeds. This could be attributed to mold development during the stratification period in the laboratory chambers since increased mold growth was observed on the two-month cold-stratified seeds of *A. marocana*. The same problem has been reported in germination studies involving seeds of other members of the genus *Abies* [40,44,46,48].

Most *Abies* species exhibit some degree of seed dormancy, and stratification is required to improve germination in terms of capacity and/or speed [25]. In the present study, the detected response to temperature and CS confirms the existence of some degree of dormancy in *A. marocana* seeds. CS is regarded as the most important treatment for breaking dormancy in the seeds of many species of the temperate zone [37,49,50,51]. It is known that when CS reduces the temperature requirement for germination or increases the speed of germination, the species should be listed [after 37] as having physiological dormancy rather than being with non-dormant seeds. 

Temperature is an important environmental factor that regulates seed germination in many plant species [52]. The Talassemtane forest, where *A. marocana* occurs naturally, in Morocco receives an average precipitation of 1500 mm annually [5,6]. In our study, the GIS-derived ecological profile of *A. marocana* informed that the mean annual precipitation in the species range is 1024.81 ± 54.99 mm. Previous studies focusing on niche modelling report that droughts, the average temperature of the warmest quarter, the maximum temperature of the warmest month, the annual average temperatures, and the annual average precipitation are critical abiotic factors for this species [9,10]. Obviously, *A. marocana* has developed specific adaptations and a survival strategy to prevent seed germination during unfavorable environmental conditions for seedling growth, such as cold or dry periods. Cones of *A. marocana* ripen in autumn with subsequent seed dispersal in mid-autumn or early winter [9]. According to the results of this study, *A. marocana* seeds germinated without any CS; however, their germination started with a month-long delay at temperatures from 15–25 °C (see Figure 4). Moreover, at 10 °C, the germination started after the 50th day. Based on the results of this study, in vivo germination in the wild habitats does not seem to happen during October and November or early winter because the temperatures recorded in the distribution area of *A. marocana* are quite lower than their preferable temperature for the initiation of seed germination (15 °C for the germination of non-stratified seeds in laboratory conditions). According to the GIS-derived ecological profile of *A. marocana* (Figure 3), the mean monthly air temperatures (not ground or below-ground temperatures) for October, November, and December are 12.55, 9.35, and 6.24 °C, respectively. In parallel, in the wild habitats of *A. marocana,* the increased precipitation over the winter months (mean precipitation from 74.16 mm in October to 123.22 mm in December and 171.98 mm in March), in combination with the low temperatures during this period (see Figure 3), probably create the ideal conditions for natural seed stratification. As inferred by the germination results of cold-stratified *A. marocana* seeds, their exposure to humid and cold conditions during wintertime, which are the appropriate conditions for dormancy-breaking, results in a widening of the temperature range over which germination actually occurs in spring. Subsequently, the seeds can probably germinate in early spring when the average mean temperatures are still relatively low (7.96 °C and 11.52 °C in March and April, respectively) and the soil moisture is high or progressively close to the favorable germination temperature detected in germination tests (15 °C).

## 4. Materials and Methods

### 4.1. Multifaceted Evaluation in Economic Sectors

The evaluation of the potential of *A. marocana* in specific economic sectors (Level I evaluation) was overviewed based on twenty attributes assessing its ornamental-horticultural potential [18], (ii) seven attributes assessing its agro-alimentary potential [27], and (iii) nine attributes assessing its potential in the medicinal-cosmetic sector [28]. A multidisciplinary scientific consortium, including 13 experienced scientists, was engaged to develop a new methodological scheme for the multifaceted evaluation of target NUPs, deciding collectively after a case-by-case examination and consultation the following issues: individual attributes per economic sector to be used for the evaluation; typology of attributes used for evaluation (sector-specific or inter-sectorial); selection of data sources to be used for documentation (one to four types per selected attribute); scaling per attribute (two-fold to seven-fold), and directionality for the scoring of different attributes (possible scores and value definitions based on the quality and quantity of the extant information retrieved). This methodological scheme is fully described in detail (e.g., individual scores per attribute) along with guidelines and examples of scorings for 399 local endemic taxa of three Mediterranean regions (Crete, Mediterranean Coast–Rif, and Tunisia) in previous studies of our own for the ornamental sector [18], the agro-alimentary sector [27], and the medicinal-cosmetic sector [28]. The evaluation of the potential of *A. marocana* in specific economic sectors (Level I evaluations) was overviewed based on twenty attributes assessing its ornamental-horticultural potential [18], (ii) seven attributes assessing its agro-alimentary potential [27], and (iii) nine attributes assessing its potential in the medicinal-cosmetic sector [28]. After the scoring of individual attributes, the sum of scorings for all attributes per economic sector was calculated and expressed as the relative percentage (%) of the maximum possible score that could be generated in each sector [18,27,28]. In this way, the relevant data and information sourced from widely scattered sources were assessed to comprehensively illustrate the relative potential of *A. marocana* in different economic sectors.

Feasibility evaluation of *A. marocana* (Level II evaluation) involved point-scoring of 12 selected attributes, considered either as prerequisites of common interest across various economic sectors (eight attributes), or as unique identity elements or special features (four attributes) to be exploited in terms of product branding and marketing [18]. The designated readiness timescale evaluation for the sustainable exploitation for *A. marocana* (Level III evaluation) involved the completion of eight criteria, based on SWOT (Strengths, Weaknesses, Opportunities, Threats) and gap analyses. These evaluations were sourced from previous research or our own [18] and are presented herein in detail for *A. marocana*.

### 4.2. Distribution Mapping and GIS Ecological Profiling

**Figure 5:**
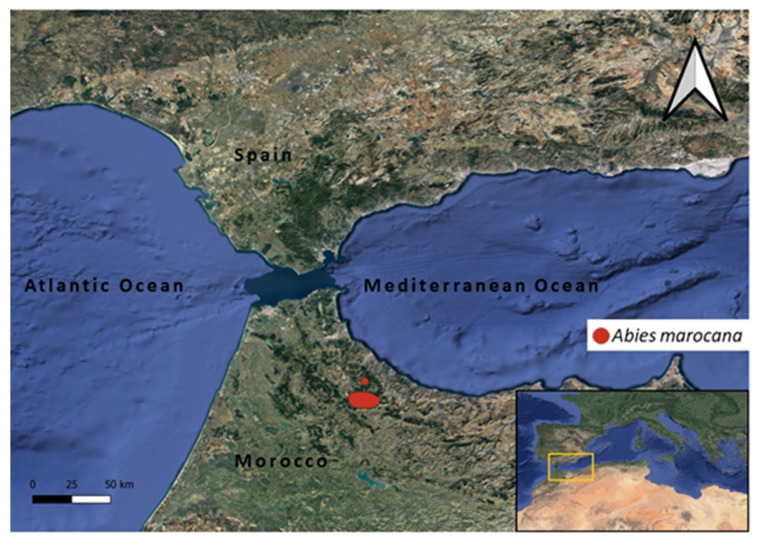
Natural global distribution range of the threatened local endemic Moroccan fir (*Abies marocana*) based on 313 occurrence records [9,53,54].

In total, 313 natural distribution points (occurrence records) of current *A. marocana* wild-growing populations were taken from previously published studies [9,53,54]. Based on the natural distribution range of *A. marocana* in Morocco (Figure 5), respective historical climate data of pixel size 30 sec was downloaded from the WorldClim website (https://www.worldclim.org/data/worldclim21.html, accessed on 26 November 2021) regarding minimum, maximum, and average temperatures and precipitation, as well as the respective values regarding 19 bioclimatic variables, i.e., Annual Mean Temperature (Bio_1), Mean Diurnal Range (Bio_2), Isothermality (Bio_3), Temperature Seasonality (Bio_4), Max Temperature of Warmest Month (Bio_5), Min Temperature of Coldest Month (Bio_6), Temperature Annual Range (Bio_7), Mean Temperature of Wettest Quarter (Bio_8), Mean Temperature of Driest Quarter (Bio_9), Mean Temperature of Warmest Quarter (Bio_10), Mean Temperature of Coldest Quarter (Bio_11), Annual Precipitation (Bio_12), Precipitation of Wettest Month (Bio_13), Precipitation of Driest Month (Bio_14), Precipitation Seasonality (Bio_15), Precipitation of Wettest Quarter (Bio_16), Precipitation of Driest Quarter (Bio_17), Precipitation of Warmest Quarter (Bio_18), and Precipitation of Coldest Quarter (Bio_19). The following layers were imported in the GIS environment: 

(a) WorldClim version 2.1 [55], containing minimum, maximum, and average temperatures (°C) as well as precipitation values (mm) and data for 19 bioclimatic variables for every month derived from 1970–2000, with a raster resolution of 1 km^2^;

and (b) an *A. marocana* distribution raster file, including 313 occurrence records [9,53,54] (Figure 5).

### 4.3. Seed Collection and Storage

**Figure 6:**
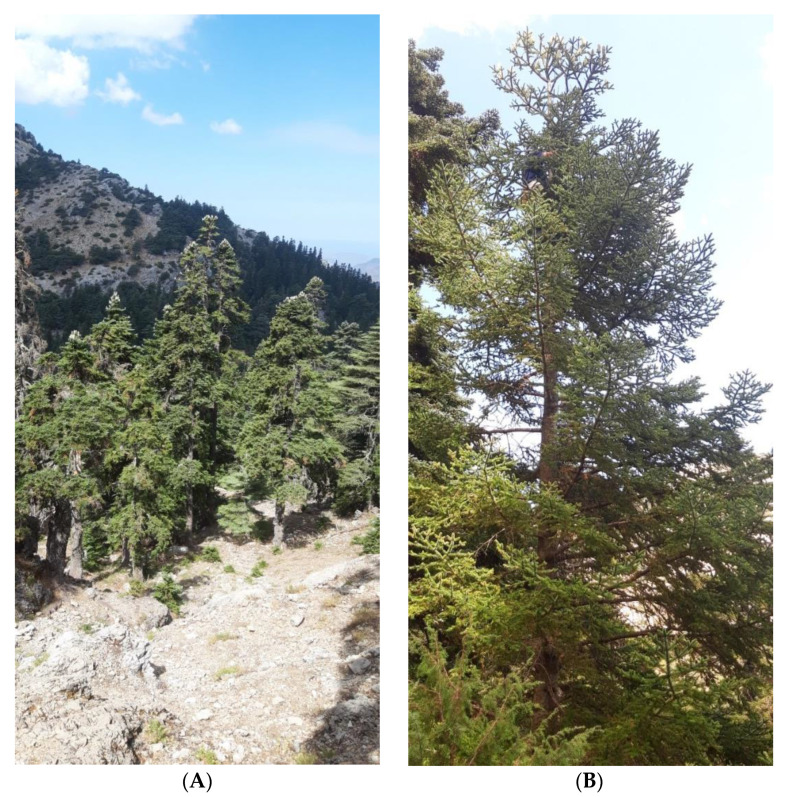

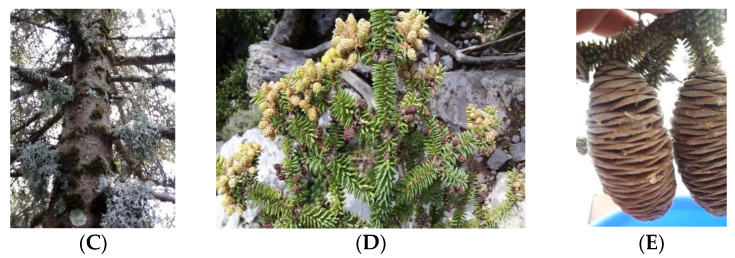
(**A**) Forests and habitat of *Abies marocana* in Talassemtane National Park of the Rif region in Morocco; (**B**) Habitat of a mature *A. marocana* individual; (**C**) Trunk and ramification of a random *A. marocana* mature individual; (**D**) Male inflorescences; (**E**) Ripe female cones with seeds.

**Figure 7:**
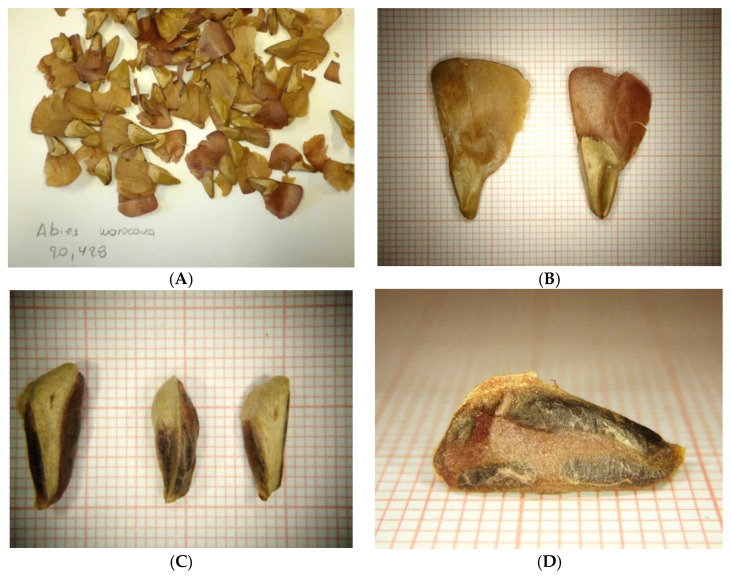
Seed morphology of *Abies marocana*: (**A**) Extracted seeds from cones; (**B**) Size variability of winged seeds; (**C**) Size variability of de-winged seeds; (**D**) Lateral view of de-winged seed.

On 31 August 2019, mature cones of *A. marocana* were collected by hand from random wild-growing individuals in natural habitats of the Talassemtane National Park, Rif, Morocco (035°07′02″ N, 05°08′00″ W and altitude 1539 m a.s.l). The species habitat was characterized by the presence of limestone rock outcrops on a steep slope (Figure 6). During collection, every paper bag with cones and seeds was labeled separately (taxon, date and location). After harvesting, the cones were stored in paper bags to allow for gradual desiccation. To extract the seeds in the laboratory, the cones were fragmented by hand and then the seeds were manually separated from the scales and cone axes (Figure 7). The separated seeds were de-winged by hand and then stored in glass containers in a laboratory refrigerator (3–5 °C).

Then, to be gradually dehumidified, the seeds were deposited for 12 months in the Seed Bank of the Institute of Plant Breeding and Genetic Resources, Agricultural Organization Demeter, in Thessaloniki, Greece, at 5 °C in a walk-in fridge with low relative humidity (25%). Prior to the seed transfer that took place in the frame of the MULTI-VAL-END project (“Multifaceted Valorisation of single-country Endemic plants of Crete, Greece, Tunisia and Rif, Morocco for sustainable exploitation in the agro-alimentary, horticultural-ornamental and medicinal-cosmetic sectors”; ARIMNet2), a MTA (Mutual Transfer Agreement) was officially signed by the legal bodies of the donor and recipient parties to ensure the endorsement of provisions of the Nagoya Protocol, as imposed by the EU Regulation 511/2014. Then, the taxonomically identified stored seedlot of *A. marocana* (Figure 6) obtained a unique IPEN (International Plant Exchange Network) accession number MA-1-BBGK-20,428 (identifier and passport data illustrating origin, restrictions of use, storage institution, year of collection, and identity number), and it was photographed using a Ricoh WG-6 camera with an incorporated LED ring light.

### 4.4. Seed Treatment

**Figure 8:**
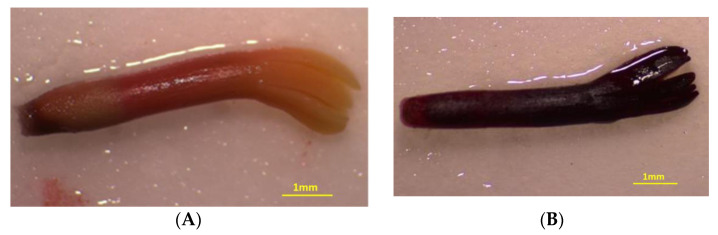
Non-viable (**A**) and viable (**B**) embryos during viability examination of *Abies marocana* seeds.

Germination experiments were initiated in January 2021 and they were conducted in the Laboratory of Horticulture, School of Agriculture, Aristotle University of Thessaloniki, Greece. Before the experiments, a random sample of 100 seeds of *A. marocana* (four replications of 25 seeds) was subjected to a tetarazolium test in order to estimate seed viability. Pre-moistened seeds were cut longitudinally beside the embryo, and then were immersed in a 1% solution of 2,3,5-triphenyl tetrazolium chloride for 18 h in the dark [38]. Subsequently, the staining of cut seeds was examined (Figure 8).

In order to determine the effect of cold stratification (CS) on germination, the seeds were mixed with moist sterilized river sand in plastic containers and were stratified for one or two months at 2–4 °C in separate plastic containers with 400 seeds each. In addition, non-stratified seeds (0 months) were subjected to germination. 

### 4.5. Germination Tests

**Figure 9:**
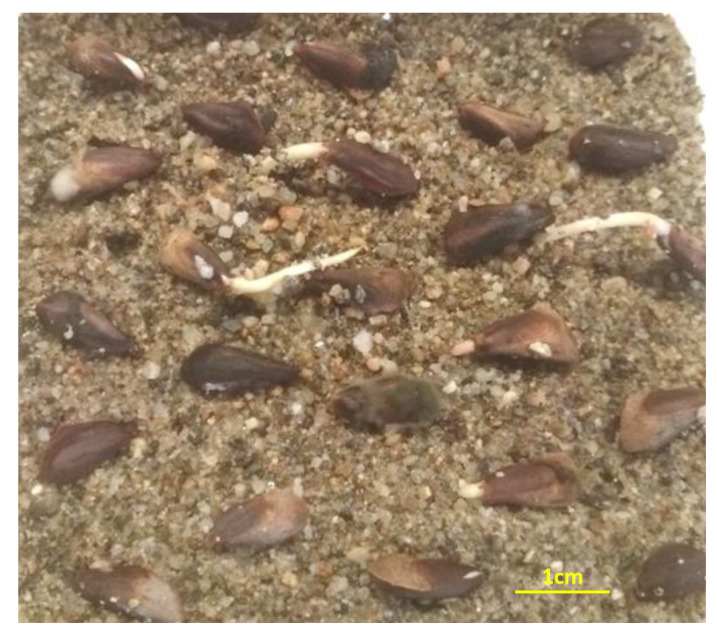
Germinated seeds of *Abies marocana* with evident radicle protrusion.

The non-stratified as well as the stratified seeds were placed for germination in growth chambers at the end of each CS period, and their germination response to four constant temperatures (10, 15, 20, and 25 °C) was evaluated. Experiments were maintained in a CRW-500SD growth chamber (Chrisagis, Athens, Greece) with relative humidity (RH) at 75 ± 1% and a light intensity of 82 μmol m^−2^ s^−1^ at the culture level from cool-white fluorescent tubes. The selection of temperature intervals was facilitated by the GIS ecological profile generated for *A. marocana* (see Section 2.2), based on 313 occurrence records located in nearby regions. For each treatment and temperature, there were four replications of 25 seeds. The seeds were placed on sterilized river sand in 12-cm glass Petri dishes, moistened with distilled water. The Petri dishes were randomly arranged on the shelves of the growth chambers under a 12-h light/12-h dark photoperiod regime, and were watered with distilled water for the whole experimental period. Attention was given to watering so as to avoid drying, as well as excess moisture, in the substratum (sand). The amount of water required to moisten the sand was not constant, and depended on the incubation temperature. Germinated seeds were counted each week for a period of 13 weeks. A seed was considered as germinated when an at least 2-mm-long radicle had emerged through the seed coat (Figure 9). Finally, for each temperature of each CS period, the germination percentage (GP) and the mean germination time (MGT) were calculated as the average of the four replications. The MGT was calculated for each replication per treatment, according to the following equation:

MGT = Σ(Dn)/Σn

where n is the number of seeds which germinated on day D, counted from the beginning of the test [56].

### 4.6. Statistical Analysis 

The experimental design was a completely randomized design with two factors. The factors were the duration of CS and the incubation temperature (3 × 4 factorial design). The GP data was transformed to arc-sine square root values before analysis [57,58]. The transformed data, as well as the MGT data, were checked for normality and homogeneity of variances, and they were analyzed using the ANOVA method [58] in the frame of the general linear model (GLM), while the comparisons of the means were performed using the R-E-G-WQ test [59,60]. All statistical analyses were carried out using SPSS 21.0 (SPSS, Inc., Armonk, New York, USA).

## 5. Conclusions

To date, there are only non-coordinated efforts for the ex-situ conservation of *Abies marocana* in five foreign countries, but no ex-situ conservation is offered in Moroccan facilities [20]. This fact is coupled with commercial demand, as illustrated by the global internet plant trade [21]. The current research explored comprehensively and documented substantially the existing potential of *Abies marocana* in different economic sectors (ornamental-horticultural, medicinal-cosmetic, and agro-alimentary), and overviewed the feasibility and readiness timescale regarding value chain creation for its sustainable exploitation. Furthermore, this study investigated the climatic conditions in which its wild-growing populations thrive naturally in Morocco, and revealed the germination requirements of *A. marocana* seeds (effects of temperature and cold stratification). Thus, the findings of this study fill in extant research gaps, may contribute to in situ and ex situ conservation strategies, and can facilitate the sustainable exploitation of this emblematic local endemic plant of northern Morocco. 

## Figures and Tables

**Table 1:** Significance of factors and their interaction on the germination percentage and on the mean germination time of *Abies marocana* seeds, as estimated by ANOVA.

Attributes	Factors	Sum of Squares	df	Mean Square	F	Sig.
	Cold Stratification (CS)	250.98	2	125.49	6.35	0.004
Germination Percentage (GP)	Temperature (T)	367.82	3	122.60	6.20	0.002
	CS * T	380.42	6	63.40	3.21	0.013
	Cold Stratification (CS)	14,770.65	2	7385.33	677.24	0.000
Mean Germination Time (MGT)	Temperature (T)	2965.46	3	988.49	90.64	0.000
	CS * T	563.41	6	93.90	8.61	0.000

**Table 2:** Interaction of the factors “cold stratification” and “temperature incubation” on germination percentage and the mean germination time of *Abies marocana* seeds.

Cold Stratification(Months)	Incubation Temperature			
	10 °C	15 °C	20 °C	25 °C
Germination percentage (% ± S.D.)				
0	12.00 b ^1^ B ^2^ ± 5.66	35.00 a A ± 6.83	19.00 ab A ± 6.83	21.00 ab A ± 3.83
1	27.00 a A ± 6.83	32.00 a A ± 8.64	26.00 a A ± 6.93	23.00 a A ± 6.00
2	24.00 a AB ± 6.53	22.00 a A ± 5.16	16.00 a A ± 5.66	15.00 a A ± 6.83
Mean Germination Time (days ± S.D.)				
0	74.26 a ^1^ A ^2^ ± 4.50	50.15 b A ± 2.40	52.77 b A ± 3.70	48.18 b A ± 4.62
1	38.13 a B ± 5.99	17.72 b B ± 1.34	14.35 b B ± 1.01	14.75 b B ± 0.96
2	22.83 a C ± 3.55	18.35 ab B ± 2.48	15.46 b B ± 2.21	12.69 b B ± 2.54

^1^ Within a row, percentages are statistically different at *p* < 0.05 when they do not share a common small letter. ^2^ Within a column, percentages are statistically different at *p* < 0.05 when they do not share a common capital letter. The comparisons were made using the R-E-G-WQ test.

## Data Availability

The data presented in this study are available on request from the corresponding authors.
